# Identification of C2H2-ZF binding preferences from ChIP-seq data using RCADE

**DOI:** 10.1093/bioinformatics/btv284

**Published:** 2015-05-06

**Authors:** Hamed S. Najafabadi, Mihai Albu, Timothy R. Hughes

**Affiliations:** ^1^Donnelly Centre for Cellular and Biomolecular Research, University of Toronto, Toronto, ON, Canada,; ^2^Department of Molecular Genetics, University of Toronto, Toronto, ON, Canada and; ^3^Canadian Institute for Advanced Research, Toronto, ON, Canada

## Abstract

**Summary:** Current methods for motif discovery from chromatin immunoprecipitation followed by sequencing (ChIP-seq) data often identify non-targeted transcription factor (TF) motifs, and are even further limited when peak sequences are similar due to common ancestry rather than common binding factors. The latter aspect particularly affects a large number of proteins from the Cys_2_His_2_ zinc finger (C2H2-ZF) class of TFs, as their binding sites are often dominated by endogenous retroelements that have highly similar sequences. Here, we present recognition code-assisted discovery of regulatory elements (RCADE) for motif discovery from C2H2-ZF ChIP-seq data. RCADE combines predictions from a DNA recognition code of C2H2-ZFs with ChIP-seq data to identify models that represent the genuine DNA binding preferences of C2H2-ZF proteins. We show that RCADE is able to identify generalizable binding models even from peaks that are exclusively located within the repeat regions of the genome, where state-of-the-art motif finding approaches largely fail.

**Availability and implementation:** RCADE is available as a webserver and also for download at http://rcade.ccbr.utoronto.ca/.

**Supplementary information:** Supplementary data are available at *Bioinformatics* online.

**Contact:** t.hughes@utoronto.ca

## 1 Introduction

Chromatin immunoprecipitation followed by sequencing (ChIP-seq) is the most widely used method for mapping the genomic regions that are associated with transcription factors (TFs) (ENCODE Project Consortium, 2012). Identification of direct TF binding sites from ChIP-seq data is an essential step for decoding the molecular mechanisms that underlie the regulatory programs dictated by TFs, and understanding how genetic changes can affect these programs. In the absence of orthogonal information on DNA binding preferences of TFs, such as *in vitro* binding data, achieving this goal primarily depends on inference of a binding model (such as a DNA ‘motif’) from the ChIP-seq data. Current approaches for motif finding from ChIP-seq data almost exclusively rely on the assumption that the genomic regions associated with a particular TF have diverse sequences except at the sites that are directly bound by the TF, where the sequences are converged to match the TF binding preference. However, this assumption is violated in many cases, such as when the ChIP-seq peaks are dominated by binding sites of the interacting partners of the TF of interest, represent targets of multiple cooperative regulatory factors, and/or are enriched for repetitive DNA sequences such as endogenous retroelements (EREs).

Binding to EREs with similar sequences particularly affects the ability of motif finding approaches for identification of DNA binding preferences of the Cys_2_His_2_ zinc finger (C2H2-ZF) class of TFs, which is by far the largest class of TFs in most vertebrates. The C2H2-ZF proteins constitute almost half of all human TFs, and almost half of them bind primarily to EREs (Najafabadi *et al.*, 2015). As a result, the motifs identified from the genomic regions that they bind often reflect the sequence homology among different instances of the associated ERE type, rather than the genuine binding preference of the C2H2-ZF protein. An alternative is to directly predict the binding preferences of C2H2-ZF TFs from their protein sequences. However, these predictions are often inaccurate (Gupta *et al.*, 2014; Najafabadi *et al.*, 2015; Persikov and Singh, 2014). In addition, not all of the C2H2-ZF domains within a protein participate in DNA binding at the same time, further complicating the task of predicting DNA preference from protein sequence.

To address these issues, we present recognition code-assisted discovery of regulatory elements (RCADE), which combines predictions from a recent recognition code of C2H2-ZFs (Najafabadi *et al.*, 2015) with motif optimization based on ChIP-seq data to overcome limitations associated with current approaches, and also to identify regions of the C2H2-ZF protein that engage in DNA-binding.

## 2 Methods

**Fig. 1.**
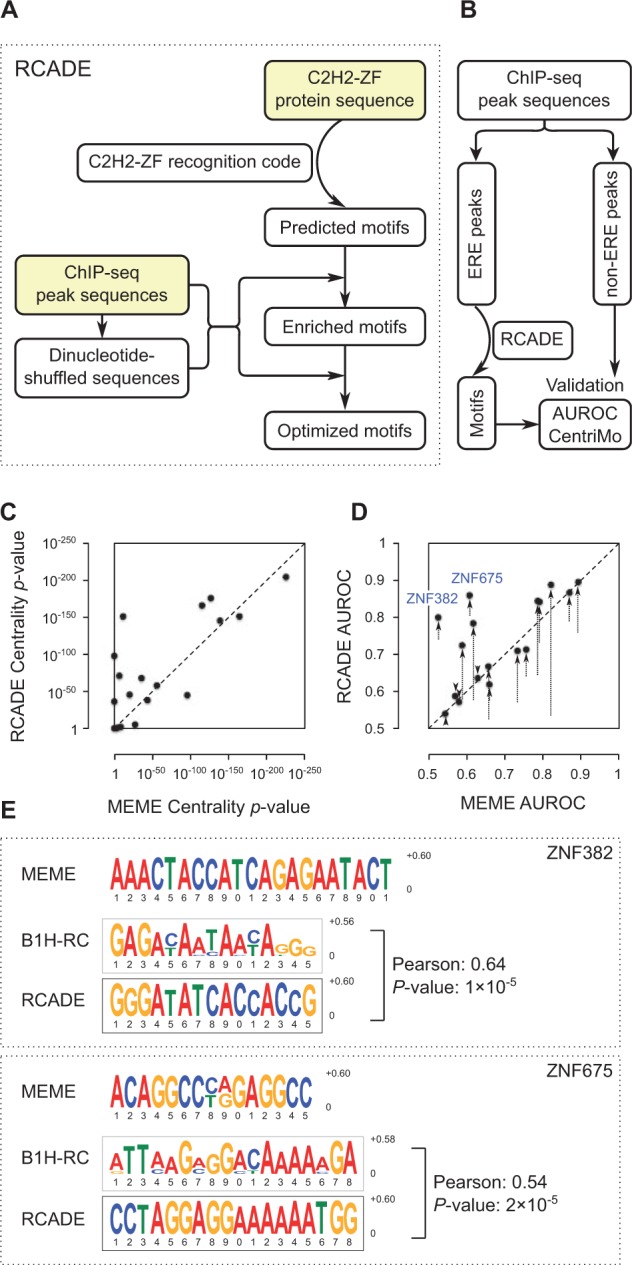
RCADE workflow and benchmarking results. (**A**) RCADE starts by predicting a set of motifs from the target C2H2-ZF protein sequence, using a previously published bacterial-one-hybrid assay-based recognition code, or B1H-RC (Najafabadi *et al.*, 2015), which are evaluated against the ChIP-seq peak sequences to identify significantly enriched motifs, and are then iteratively optimized. (**B**) Benchmarking workflow for evaluation of RCADE. The peak sequences were divided into two sets of ERE-overlapping and non-ERE peaks. The ERE-overlapping peaks for each protein were used for motif discovery using RCADE, and the motifs were validated using non-ERE peaks. (**C,D**) Validation results for 18 ERE-binding proteins. The arrows show the improvement in the AUROC of RCADE motifs compared with seed B1H-RC motifs. (**E**) Example motifs for two proteins that show the largest difference between RCADE and MEME validation results. The top-scoring MEME motif is shown for each protein, followed by the top-scoring motif that is directly predicted from protein sequence using the B1H-RC, and the RCADE optimized motif. The Pearson similarity of the B1H-RC and RCADE motifs was calculated as described previously (Najafabadi *et al.*, 2015)

RCADE examines the C2H2-ZF domains within a protein to identify stretches of adjacent zinc fingers, or zinc finger ‘arrays’, whose predicted binding sites (Najafabadi *et al.*, 2015) are enriched in ChIP-seq peaks relative to dinucleotide-shuffled sequences, indicating direct DNA binding. Then, RCADE optimizes the motifs to discriminate between the real and shuffled sequences (Fig. 1A). Briefly, for each predicted seed motif, RCADE identifies the sequences with the largest motif scores, and constructs a new Position Weight Matrix (PWM) by aligning the motif hits in these sequences, repeating this procedure until the PWM converges. The top-scoring optimized PWM is reported, along with the zinc fingers that are predicted to contribute to DNA-binding. The optimized motifs are almost always significantly similar to the original seed motifs, indicating that the optimization procedure does not depart drastically from the starting point. The RCADE algorithm is shown in more detail in Supplementary Figure S1.

We have implemented RCADE in a webserver, which can be accessed at http://rcade.ccbr.utoronto.ca/. The input and output of the RCADE webserver are described in Supplementary Figure S2.

## 3 Benchmarking

To evaluate the performance of RCADE in identifying correct motifs from highly similar sequences, we applied it to the set of ChIP-seq data for all the 18 human proteins shown to bind to EREs in a previous study (Najafabadi *et al.*, 2015). We identified the 500 most enriched ERE-overlapping peak summits as well as the 500 most enriched non-ERE peak summits for each dataset, and trained the RCADE motifs exclusively on the ERE-overlapping peaks. Since EREs have highly similar sequences due to common ancestry, it is very difficult to distinguish the correct TF motifs from unrelated enriched sequences, and therefore, most current motif finding approaches are expected to perform poorly. We used MEME (Bailey and Elkan, 1994) for comparison, as it is one of the most widely used motif finding methods. The motifs that were trained on the ERE-overlapping peaks were evaluated using non-ERE peaks (Fig. 1B), to confirm that RCADE does not overfit the motifs on the EREs. Non-ERE sequences are not expected to be similar due to common ancestry, and therefore, motif enrichment is an indicative of biological relevance.

The RCADE motifs generally showed considerably better enrichment at the center of the non-ERE peaks compared with MEME motifs, as evaluated by CentriMo (Bailey and Machanick, 2012) (Fig. 1C). Furthermore, many RCADE motifs are significantly better than MEME motifs at distinguishing non-ERE peaks from dinucleotide-shuffled sequences (Fig. 1D). Two prominent examples of such motifs are shown in Figure 1E. Further validation results are shown in Supplementary Figures S3–S7. We note that in addition to its utility for motif derivation, RCADE pinpoints the C2H2-ZF domains that engage DNA. While RCADE currently supports only the C2H2-ZF class of TFs, its concept can also be applied to other TF classes as long as a suitable recognition code exists.

## Funding

This work was supported by grants from the Canadian Institutes of Health Research (MOP-77721 and MOP-111007), and funding from Canadian Institute for Advanced Research to T.R.H. H.S.N. was supported by a Canadian Institutes of Health Research Banting Fellowship.

*Conflict of Interest*: none declared.

## Supplementary Material

Supplementary Data
