# Supplementary material for: Identification of C2H2-ZF binding preferences from ChIP-seq data using RCADE
Source: Bioinformatics. 2015 May 6;31(17):2879–81. doi: 10.1093/bioinformatics/btv284 (PMC4547615; doi:10.1093/bioinformatics/btv284)
Supplement: Supplementary Data [file supp_31_17_2879__index.html]

Identification of C2H2-ZF binding preferences from ChIP-seq data using RCADE — Identification of C2H2-ZF binding preferences from ChIP-seq data using RCADE — Identification of C2H2-ZF binding preferences from ChIP-seq data using RCADE — Supplementary Data 

# Identification of C2H2-ZF binding preferences from ChIP-seq data using RCADE

## Supplementary Data

files

- Supplementary Data - pdf file
