# Supplementary material for: Identification of C2H2-ZF binding preferences from ChIP-seq data using RCADE
Source: Bioinformatics. 2015 May 6;31(17):2879–81. doi: 10.1093/bioinformatics/btv284 (PMC4547615; doi:10.1093/bioinformatics/btv284)
Supplement: Supplementary Data [file supp_btv284_SuppFigures_v7.pdf]

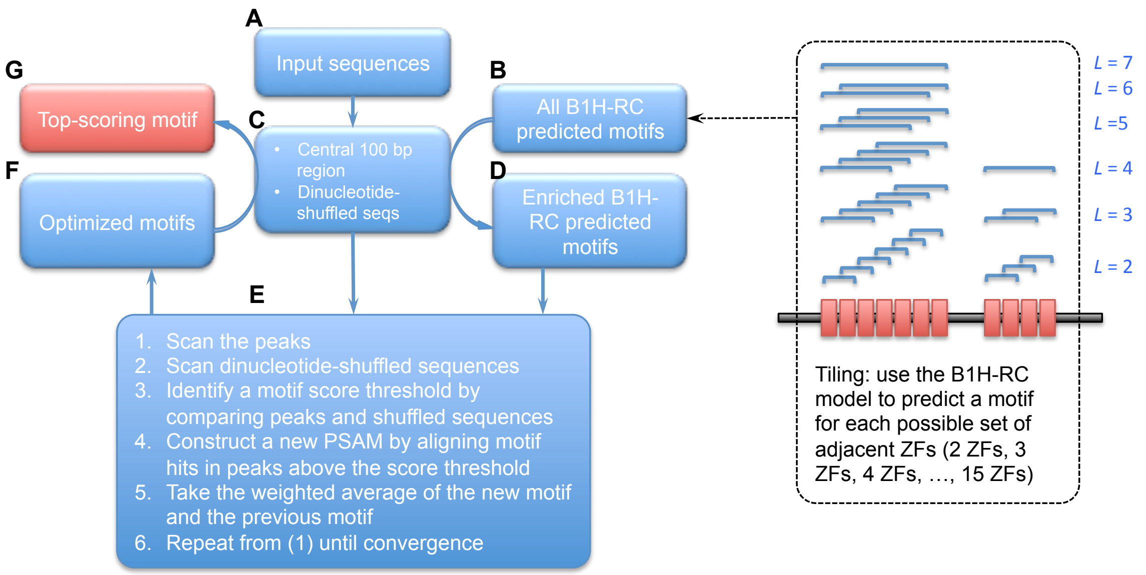

Supplementary Figure 1. The RCADE optimization algorithm. RCADE uses the C2H2 recognition code to predict the binding specificities of C2H2-ZF proteins from protein sequence, and optimizes the predicted motifs using ChIP-seq data. Since not all zinc fingers within a protein engage in binding to DNA, and therefore RCADE does not initially know which zinc fingers should be used for predicting binding specificity, RCADE tries all possible sets of adjacent zinc fingers, predicts a motif for each set using the recognition code, and optimizes the motifs independently using the ChIP-seq data. In the end, the optimized motif with the best AUROC is reported, along with the corresponding zinc finger set as the "C2H2-ZF array" that is involved in protein-DNA interaction.

The RCADE optimization module takes as input a set of ChIP-seq peak sequences (**A**), and also a set of motifs predicted by B1H-RC[1] based on the targeted C2H2-ZF protein sequence (**B**). The input peak sequences are then trimmed from two ends to retain only the central 100bp region, and a dinucleotide-shuffled version of each sequence is generated (**C**). Then, the B1H-RC predicted motifs are converted to position-specific affinity matrices (PSAMs), and are examined by scanning the original and shuffled sequences, calculating the AUROC for distinguishing original from dinucleotide-shuffled peaks as described previously[1]. Predicted motifs with AUROC p-value $< 10^{-4}$ are kept (**D**) as seeds for optimization. These may include several, potentially overlapping motifs, which are predicted from different tiling "arrays" of zinc fingers of the C2H2-ZF protein (right panel). The optimization (**E**) starts with each seed motif (PSAM), scans the original and shuffled sequences with this PSAM (**E.1**, **E.2**), and then identifies a PSAM score threshold that corresponds to the Kolmogorov-Smirnov statistic for comparison of the score distribution of original and shuffles sequences (**E.3**). Formally, it identifies a score threshold s, so that $s = \arg\max_x [\, F_{shuff}(x) - F_{orig}(x) \,]$, where F_{orig} is the empirical cumulative distribution function of PSAM scores for the original peak sequences, and F_{shuff} is the empirical cumulative distribution function for dinucleotide-shuffled sequences. In other words, the PSAM score threshold is chosen to simultaneously maximize the fraction of protein-bound sequences that are above the threshold and the fraction of shuffled sequences below this threshold. The best-scoring motif hit in each peak sequence with PSAM score above s is then identified, and these motif hits are aligned to create a new PSAM (**E.4**). The weighted average of the new and the previous PSAM is then taken (new:previous=1:4, **E.5**), and is used as the seed for the next round of optimization (**E.6**). In other words, in each round of optimization, the PSAM from the previous round is mixed with the new PSAM in order for a more gradual optimization, with the PSAM at the end of each round being 80% similar to the PSAM at the end of the previous round and 20% similar to the PSAM that is obtained by aligning motif hits. This procedure is repeated until the set of sequences used to construct the new PSAM does not change for 20 consecutive rounds, at which point the procedure is deemed to have converged. The AUROC of each optimized motif is then calculated using the original and shuffled sequences (**F**), and the motif with the largest AUROC is reported (**G**), along with sub-optimal motifs.

1 Najafabadi, H. S. *et al.* C2H2 zinc finger proteins greatly expand the human regulatory lexicon. *Nat Biotechnol* doi:10.1038/nbt.3128 (2015).

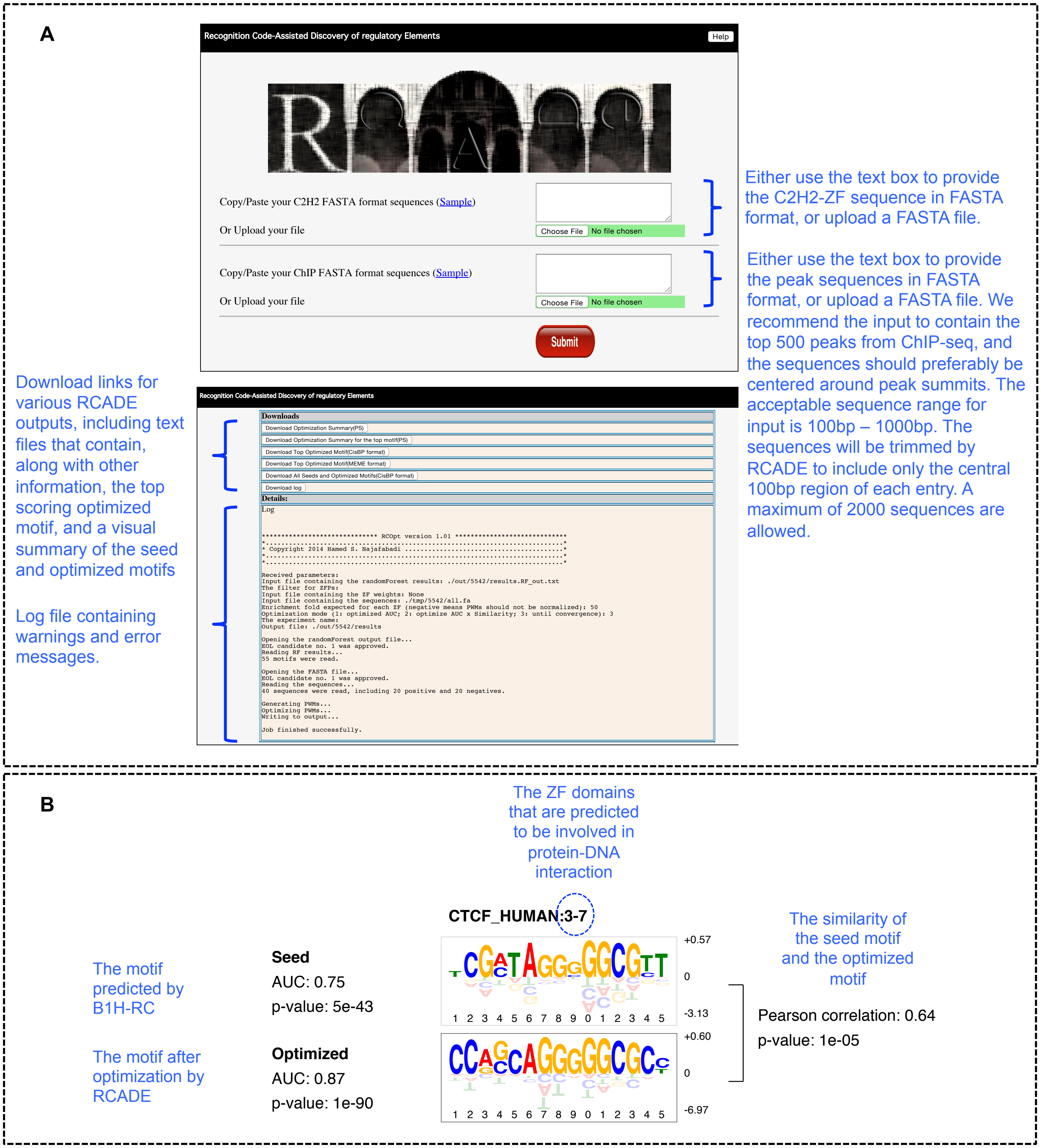

Supplementary Figure 2. (A) The RCADE webserver input page (top) and the results page (bottom). **(B)** Example RCADE output for the top-scoring PWM.

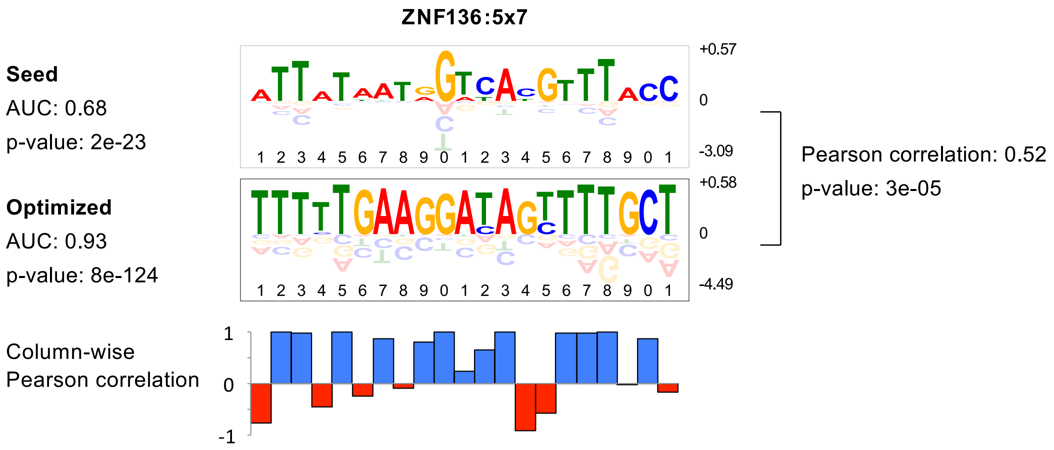

Supplementary Figure 3. Example optimization of the seed B1H-RC motif, corresponding to the longest arrow in Figure 1D. The optimized RCADE motif is highly similar to the seed B1H-RC motif. However, mostly by changing a minority of positions (red bars at the bottom graph), RCADE is able to obtain optimized motifs that may largely outperform the seed motif. The AUC values in this figure correspond to the performance of the motifs on ERE peaks.

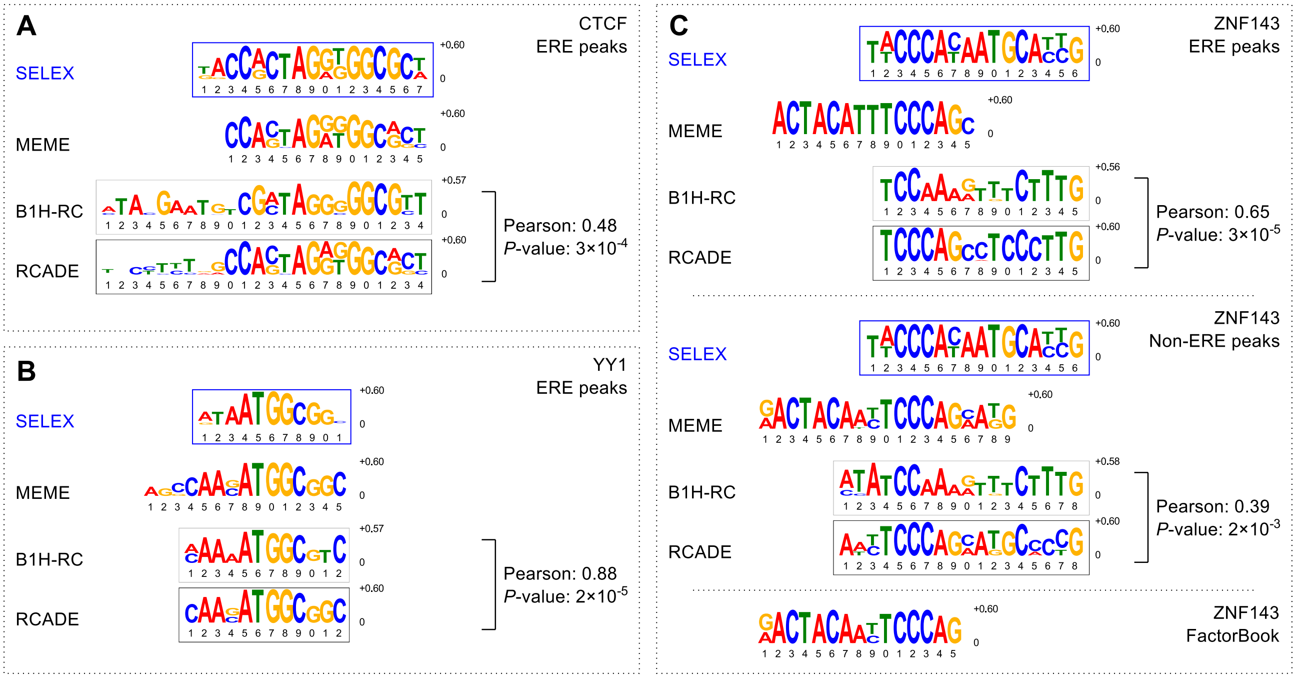

Supplementary Figure 4. Performance of RCADE for three C2H2-ZF proteins with available *in vitro* binding preferences. CTCF, YY1, and ZNF143 were chosen based on (*i*) partial binding to EREs as reported previously[2], (*ii*) presence of C2H2 zinc fingers, and (*iii*) availability of *in vitro* motifs. Peak sequences from the ENCODE Regulation "Txn Factor" track of the UCSC Genome Browser[3] were used for motif discovery. While both MEME and RCADE motifs that are obtained from ERE peaks for CTCF and YY1 are similar to the *in vitro* motif obtained by SELEX[4] (**A**, **B**), the MEME motifs for ZNF143, from either ERE or non-ERE peaks, as well as the motif reported in FactorBook[5] only partially overlap the SELEX motif (**C**). In contrast, RCADE is able to identify motifs that almost entirely cover the SELEX motif, from both ERE and non-ERE peak sets, suggesting that the *in vivo* binding preference of ZNF143 is similar to its *in vitro* binding preference. The Pearson similarity of the B1H-RC and RCADE motifs and the associated *p*-value are calculated as described previously[1], reflecting the Pearson correlation of the logarithm of the PSAM scores of each motif pair across all possible sequences that have the same length as the motif, and the probability that a randomized version of the motifs with permuted nucleotide frequencies would obtain a better Pearson correlation.

2 Wang, J. *et al.* Sequence features and chromatin structure around the genomic regions bound by 119 human transcription factors. *Genome research* **22**, 1798-1812, doi:10.1101/gr.139105.112 (2012).
3 Rosenbloom, K. R. *et al.* The UCSC Genome Browser database: 2015 update. *Nucleic acids research*, doi:10.1093/nar/gku1177 (2014).
4 Jolma, A. *et al.* DNA-binding specificities of human transcription factors. *Cell* **152**, 327-339, doi:10.1016/j.cell.2012.12.009 (2013).
5 Wang, J. *et al.* Factorbook.org: a Wiki-based database for transcription factor-binding data generated by the ENCODE consortium. *Nucleic acids research* **41**, D171-176, doi:10.1093/nar/gks1221 (2013).

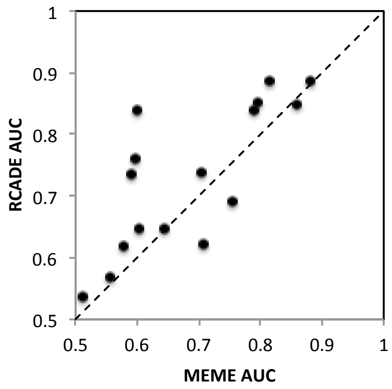

Supplementary Figure 5. Comparison of the performance of RCADE and MEME motifs in distinguishing binding sites from random genomic fragments. Similar to Figure 1D, each dot represents one protein, and the axes represent the area under the ROC curve for the motif obtained for that protein using either MEME or RCADE. Motifs are learned from ERE sequences, and the AUCs are calculated using non-ERE sequences. While Figure 1D shows the AUC for distinguishing the top 500 non-ERE peaks from dinucleotide-shuffled sequences, this figure shows the AUC for distinguishing the top 500 non-ERE peaks from 500 random human genomic regions with matching length and dinucleotide frequencies. RCADE significantly outperforms MEME ($p<0.008$, paired t-test).

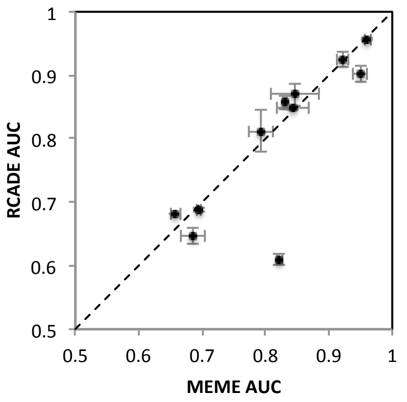

Supplementary Figure 6. Comparison of RCADE and MEME performance in identification of motifs from non-ERE regions. The top 500 peaks for each experiment were randomly split in two sets, and the motifs were learned using either RCADE or MEME from one set (training), and the AUC was determined using the other set (testing). Each data point represents one protein, with the x- and y-axes showing the average AUC for RCADE and MEME, respectively, based on two-fold cross-validation. The error bars mark the minimum and maximum AUC using different training and testing halves.

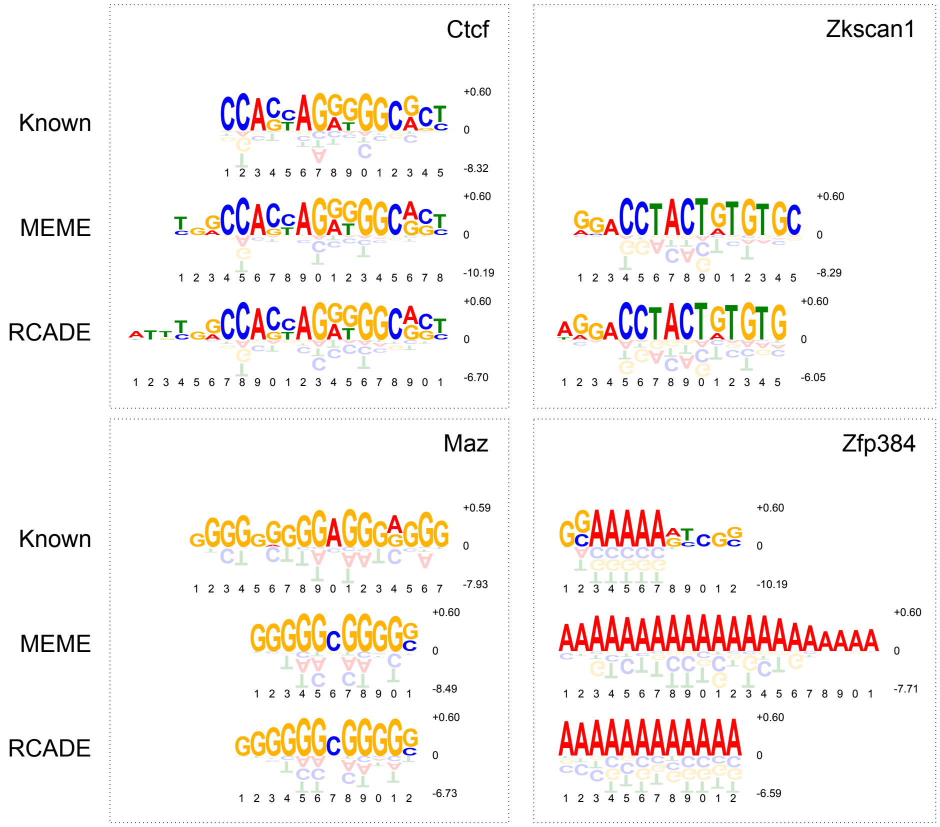

Supplementary Figure 7. RCADE is able to identify known motifs from ChIP-seq data for mouse C2H2-ZF proteins. Top 500 peaks from ChIP-seq data of C2H2-ZF proteins in mouse CH12 cells[6] were used to train motifs by MEME or RCADE. These motifs are compared to previously published motifs from mouse Ctcf[7], the human homolog of Maz[8], and the human homolog of Zfp384[8].

6 Mouse ENCODE Consortium. An encyclopedia of mouse DNA elements (Mouse ENCODE). *Genome biology* **13**, 418, doi:10.1186/gb-2012-13-8-418 (2012).

7 Chen, L., Wu, G. & Ji, H. hmChIP: a database and web server for exploring publicly available human and mouse ChIP-seq and ChIP-chip data. *Bioinformatics* **27**, 1447-1448, doi:10.1093/bioinformatics/btr156 (2011).

8 Kulakovskiy, I. V. *et al.* HOCOMOCO: a comprehensive collection of human transcription factor binding sites models. *Nucleic acids research* **41**, D195-202, doi:10.1093/nar/gks1089 (2013).
